# Enhancing Thermomechanical Strength and Thermal Stability of Poly(dicyclopentadiene) Composites through Cost-Effective Fly Ash Reinforcement for Structural and Impact Applications

**DOI:** 10.3390/polym15224418

**Published:** 2023-11-16

**Authors:** Henry A. Colorado, Wei Yuan, Juan Meza, Franklin Jaramillo, Elkin I. Gutierrez-Velasquez

**Affiliations:** 1 Composites Laboratory, Engineering School, Universidad de Antioquia (UdeA), Calle 70 No. 52-21, Medellín 050010, Colombia; 2 Department of Materials Science and Engineering, University of California, Los Angeles, CA 90095, USA; yuanwei99@gmail.com; 3 Universidad Nacional de Colombia, Calle 75 No 79A 51, Bloque M17, Medellín 050032, Colombia; jmmezam@unal.edu.co; 4 Centro de Investigación, Innovación y Desarrollo de Materiales—CIDEMAT, Facultad de Ingeniería, Universidad de Antioquia (UdeA), Calle 62 No. 52-59, Medellín 050010, Colombia; franklin.jaramillo@udea.edu.co; 5 Faculty of Engineering and Basic Sciences, Fundación Universitaria Los Libertadores, Bogotá 111221, Colombia; elkin.gutierrez@libertadores.edu.co

**Keywords:** polymer composites, reinforcements, mechanical properties, nanoindentation

## Abstract

Poly(dicyclopentadiene) (poly-DCPD) is a thermoset with potential for high-performance applications. In this research, epoxy resin was blended with different concentrations of fly ash class F particles at 0.0, 1.0, 10.0, and 50.0 wt.%, aiming to improve its use as a high-volume structural material by decreasing costs and reducing its negative environmental impact through using fly ash particles. A planetary Thinky mixer was used to initially mix the resin with the curing agent, followed by incorporating a Grubbs catalyst. The microstructures were analyzed using scanning electron microscopy (SEM), where particles were found to be homogeneously distributed over the polymer matrix. The thermomechanical behavior was evaluated via curing, compression, dynamic mechanical analysis (DMA), and thermo-gravimetric analysis (TGA). Nanoindentation tests were also conducted. Fly ash was found to decelerate the curing of the resin through the release of calcium ions that enhanced the exothermic reaction.

## 1. Introduction

High-performance resins are experiencing increasing utilization not only in traditional composite applications like aerospace [1], automotive [2], and sporting goods [3] but also in emerging areas such as energy generation, materials for extreme dynamic conditions, and construction materials. In the realm of energy generation, the demand for stronger, more durable, and recyclable materials is particularly pressing for wind power generation [4], given the sheer scale of wind turbines. Applications involving extreme dynamic conditions encompass armor and military applications [5] as well as scenarios where components are subjected to impact [6] or fatigue [7]. Construction applications extend to critical infrastructure like buildings [8], bridges [9], and even composite pipes for sewer and water systems [10]. The modern approach of patch repair using resins necessitates the use of high-strength resins [11]. As demonstrated earlier, the requirement for enhanced resins is pervasive across numerous industries, making any advancements or refinements in these materials highly applicable and valuable.

Among the various resins used, epoxy stands out as one of the most widely employed globally due to its exceptional mechanical and thermal properties [12]. Poly-DCPD [13] is another noteworthy resin, renowned for its high impact resistance and corrosion resistance. It can be further enhanced through reinforcement, like epoxy. Poly-DCPD is recognized for possessing comparable tensile strength to epoxy but with distinct advantages such as a higher glass transition temperature (Tg), a lower density, and significantly reduced water absorption. These attributes make it highly suitable for various high-performance applications.

However, when it comes to extreme dynamic conditions, such as ballistic responses, it has been reported that poly-DCPD exhibits a remarkable 300–400% improvement over structural epoxy resins [14]. This substantial difference in performance, particularly when combined with appropriate fibers, makes poly-DCPD an outstanding choice for such demanding applications.

Dicyclopentadiene (DCPD, C_10_H_12_) is a colorless liquid with a camphor-like odor that is formed from the spontaneous Diels–Alder dimerization of cyclopentadiene. DCPD is a significant component of the C5 olefin stream formed as a byproduct from the production of ethylene via steam cracking of naphtha. Several hundred million pounds (only a fraction of the total produced) of DCPD of various purity levels are recovered each year and utilized in a variety of applications, such as tackifiers for adhesives and inks, and co-monomers for polyester resins or EPDM rubbers [13,15,16,17,18].

DCPD is also polymerized through ring-opening metathesis with a ruthenium-based Grubbs catalyst to form a cross-linked polymer with high modulus, strength, and impact resistance [19,20]. Two steps are involved in this ring-opening polymerization: First, the opening of the strained cyclopentene ring with the aid of a catalyst forms the linear structure at below 40 °C. Secondly, a strong exothermal reaction assists the cross-linking at over 80 °C. Cross-linked poly-DCPD has been successfully used for truck body panels, sporting goods, and drain parts. Due to its unique properties, such as low viscosity as a monomer and high strength and toughness after polymerization, it has been extensively investigated as a self-healing agent [21]. In addition to its low cost, it has been worked with asphalt materials to reinforce pothole-patching materials [22].

The incorporation of fillers in rubber compounds, such as fly ash, has shown significant improvements in various properties, such as elongation at break, adhesion to the reinforcing steel cord, wet grip, and reduced rolling resistance. These improvements are attributed to the more effective filler dispersion and reinforcing effect of silica found in fly ash [23]. The chemical composition of fly ash, characterized by its high silica content, makes it an attractive filler for industrial elastomer applications. However, challenges remain due to the need for costly particle treatment involving heavy metal leaching and size reduction. Nevertheless, the utilization of fly ash as a feedstock for high-volume polymeric products presents a cost-effective approach to pollution prevention and material valorization [24].

In addition, research on the impact of filler content and applied voltage under DC conditions has been conducted to evaluate the ability of composite materials to store and recover electrical energy. In particular, the inclusion of fly ash has shown an improvement in energy storage and recovery efficiency for these composite materials [25]. Another study explored the influence of basalt fiber and waste marble dust on epoxy resin. The results revealed improvements in tensile, flexural, and impact properties, up to 7.5 wt.% of waste marble powder. Moreover, the Vickers hardness of the epoxy increased with different levels of waste marble powder reinforcement. In addition, the specific wear rate increased with the addition of waste marble powder up to 7.5% by weight. Scanning electron microscopy was employed to analyze the nature of fractured surface wear phenomena in these composite materials [26]. A substantial increase in impact resilience was observed, even at 50 wt.% filler ratios, after the introduction of modified brick powder, which resulted in higher heat resistance and thermal conductivity in the epoxy composite [27].

Fly ash is a byproduct of the coal combustion industry in the way of micro-hollowing particles. Fly ash has been extensively used in cement and polymer composites as hydration and set control, filler, and reinforcement. Although fly ash is extensively used in concrete, its use in high-performance resins is more valuable due to the composite costs by weight. Fly ash has been extensively used as particle reinforcement for polymer composites, with a tensile strength below 8 MPa when using a thermoplastic matrix [28], a compressive strength between 84 and 102 MPa when using an epoxy resin, and a tensile strength between 60 and 110 MPa for epoxy resin as well, in cases using up to 40 wt.% of fly ash. These results show that fly ash can be a valuable ceramic reinforcement in different polymeric matrices, which opens possibilities for poly-DCPD composites.

In addition, this research delves into the incorporation of fly ash, a natural mineral filler, into poly-DCPD resin, underscoring its commitment to environmentally friendly manufacturing practices. By leveraging the inherent attributes of fly ash, such as minimal processing requirements and the absence of organic solvents, this study aligns with the principles of sustainable materials engineering, with the primary goal of mitigating the environmental footprint associated with the production of composite materials.

In this study, fly ash particles serve as a filler for the polymeric poly-DCPD resin. The environmental friendliness inherent in this filler, as a natural mineral that does not require energy-intensive processing and does not involve additional organic solvents, underscores the environmentally friendly approach taken in this research. The manufacturing process is characterized by its simplicity, involving basic mechanical mixing and in situ polymerization techniques, which facilitates the preparation of poly-DCPD and fly ash composite materials. This research extends to examining the structure–property relationship of these particles, as well as exploring the role played by calcium ions in the curing and polymerization processes.

## 2. Materials and Experimental Methods

DCPD resin (EXP-1102, CAS Number: 64742-16-1) and Grubbs catalyst (EXP-1250) were obtained from Materia Inc., located in Pasadena (USA), and used as received (assay purity ≥ 95.0%). The DCPD resin is a low-viscosity liquid at room temperature, composed of pure DCPD and 24 wt.% trimer. A powder of second-generation Grubbs catalyst was dispersed inside mineral oil to obtain a brown-red dispersion. The catalyst-to-oil ratio used was 50:1 in weight, which corresponds to a molar ratio of 60,000:1 between the DCPD resin and the Grubbs catalyst dispersion.

Sets of four samples per composition of poly-DCPD with fly ash type F powder in different concentrations were fabricated by first mixing the resin with the curing agent of Grubbs catalyst and followed by adding the fly ash powder obtained from LaFarge (Herndon, VA, USA). Samples with different powder content were fabricated: 0.0 (neat resin), 1.0, 10.0, and 50.0 wt.% fly ash class F powder, obtained from Diversified Minerals Inc (USA) (see Table 1). The fly ash composition is presented in Table 2. The mixing process of the components was conducted in a Planetary Centrifugal Mixer (Thinky Mixer AR-250, TM, Tokyo, Japan). A mixing time of 5 min was used for all samples. This choice was based on multiple considerations. First, it serves to achieve uniform dispersion of fly ash particles in the poly-DCPD resin and Grubbs catalyst, which is essential to ensure uniform properties and improve the performance of the composite. Second, this selection considers the viscosity of the poly-DCPD resin and the characteristics of the fly ash particles. Materials with higher viscosities may require longer mixing times to achieve efficient mixing, so the decision to use a 5 min mixing duration strikes a balance between the requirement for thorough mixing and the goal of minimizing processing time and energy consumption.

Compression tests were conducted using an Instron 3382 apparatus running at a crosshead speed of 1 mm/min. For SEM examinations, the samples were mounted on an aluminum stub and sputtered in a Hummer 6.2 system (15 mA AC for 30 s), creating a thin solid film of gold. The Raman spectra of the samples were recorded using a Horiba Yvonjobin dispersive micro-Raman spectrophotometer equipped with a 632.8 nm laser (He–Ne laser, San Marcos, NJ, USA) for the excitation radiation. All the spectra were collected in the range of 3500–100 cm^−1^. Dynamic mechanical analysis (DMA) tests were conducted using a TA RSAIII dynamic mechanical analyzer at a rate of 5 °C/min. The three-point bending setup was used for all tests. All samples were rectangular bars of 2 mm in thickness, 6.0 mm in width, and 25 mm in effective length. The tests were conducted at a frequency of 1 Hz and with strain amplitude less than 3%. Thermogravimetric analysis (TGA) was performed using Perkin Elmer Pyris Diamond TG/DTA equipment. The temperature ramp was 10 °C/min. All experiments were conducted in an argon atmosphere.

Indentations were conducted using an Ibis Authority nanoindenter, which was manufactured by Fischer Cripps Labs and equipped with a Berkovich diamond indenter (Sydney, Australia). A loading–unloading cycle in closed-loop mode at 10 mN of the maximum load was scheduled for all samples under investigation. To overcome unloading creep, a dwell time of 10 s at maximum load was implemented [29]. To achieve thermal equilibrium, the samples were placed inside the instrument for 4 h in a room with constant temperature. A thermal drift rate lower than 0.05 nm/s measured at 0.015 mN (the minimum load for initial depth finding) was required before each indentation started. Each indentation experiment lasted about 30 s, so the maximum error in the measured depth due to thermal variations was about 1.5 nm out of about 500 nm. The initial contact depth was estimated using a linear interpolation of the very first initial data, followed by extrapolation to zero load as described before [30]. The area function of the indenter was calibrated with a fused silica standard sample, and all the data were analyzed using the Oliver and Pharr method [31].

## 3. Results and Analysis

**Figure 1:**
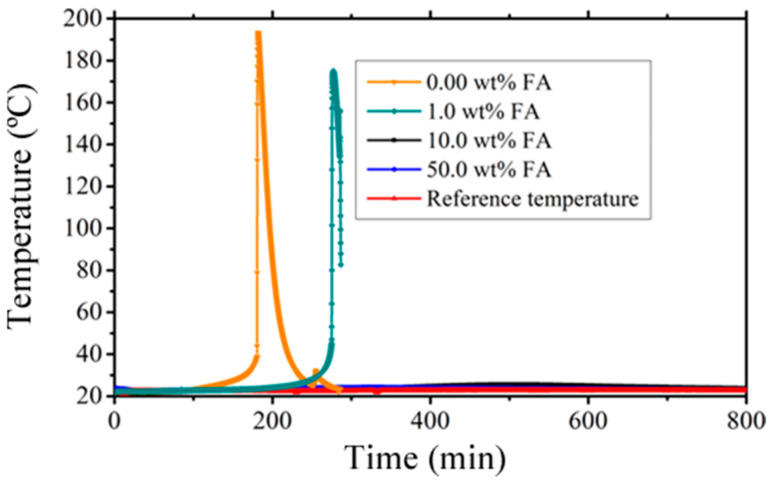
Curing experiments conducted on DCPD–fly ash composite materials.

The inclusion of only 1 wt.% fly ash (FA) led to a significant prolongation of the curing time, which was approximately 100 s longer. This effect is visually depicted in Figure 1, which illustrates that as the FA content increases, the temperature of the exothermic peak decreases from 193 to 175 °C, when compared to the pure sample (0.0 wt.%) containing 1.0 wt.% FA. This delay in reaction time agrees with previous observations in situations involving ceramic powders blended with poly-DCPD [32]. In those cases, the delay is related to the dissolution of ions that accelerate the curing process. The impact on exothermic reaction is substantial, to the extent that the peak disappears, especially at higher particle loadings, indicating a pronounced modification of the resin due to ion dissolution. However, it should be noted that air trapped in the hollow ash microspheres could also contribute to the modification of the damping properties, given the dichotomy of the mechanical properties. This phenomenon could be associated with voids and poor particle bonding, as revealed by the SEM micrographs.

**Figure 2:**
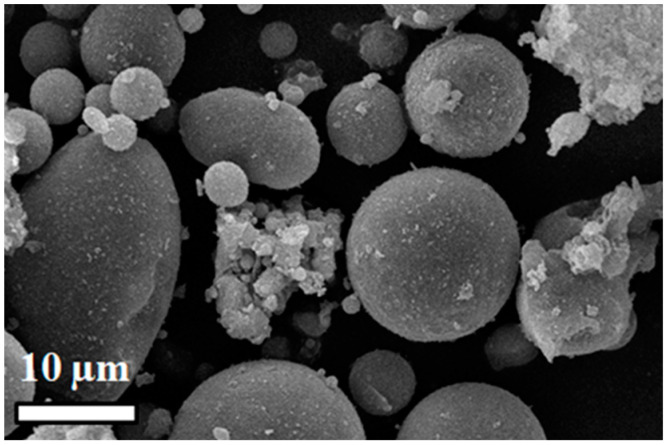
Scanning electron microscopic (SEM) image of fly ash particle morphology.

Figure 2 shows the F-class particle characteristics of the FA used, which consists predominantly of spherical particles with small amounts of amorphous particles, all of which align with the attributes of the ceramic particles as given in Table 2.

**Figure 3:**
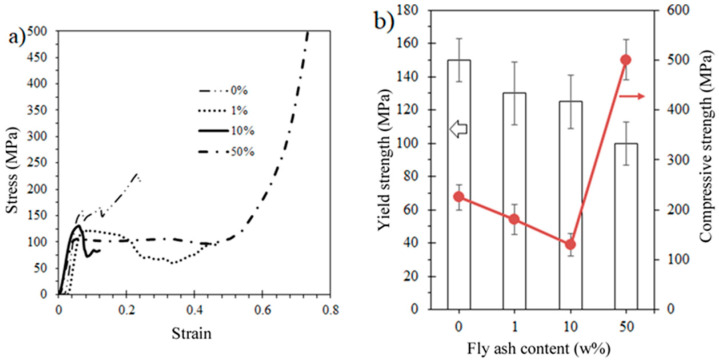
Compression test results: (**a**) selected stress–strain curves; (**b**) yielding and compressive strength values. The arrow shows the axis corresponding to the curve.

The compression tests showed that the poly-DCPD composites are quite influenced by the addition of FA (see Figure 3a), with a lot of variation in stress and strain values. Figure 3b summarizes the strength metrics as a function of fly ash loading ratio. First, as the FA content increases, the yielding point decreases as well. Then, with the ultimate strength, as the FA content increases, the compressive strength decreases; although for the highest loading at 50 wt.%, the compressive strength increases out of the scale. This has been interpreted before [32] as particle–particle touching, thus corresponding to a compressive strength fully influenced by the ceramic particles compressive among them and therefore squeezing the polymeric matrix. Indeed, at this stage, the level of damage in the sample is deemed unacceptable for most applications, with the potential exception of scenarios involving impact loading. The reduction in stress-related properties is primarily influenced by structural imperfections, such as voids. These voids are more prevalent in samples with a higher particle loading due to issues like agglomeration and inadequate mixing, resulting in reduced material homogeneity.

**Figure 4:**
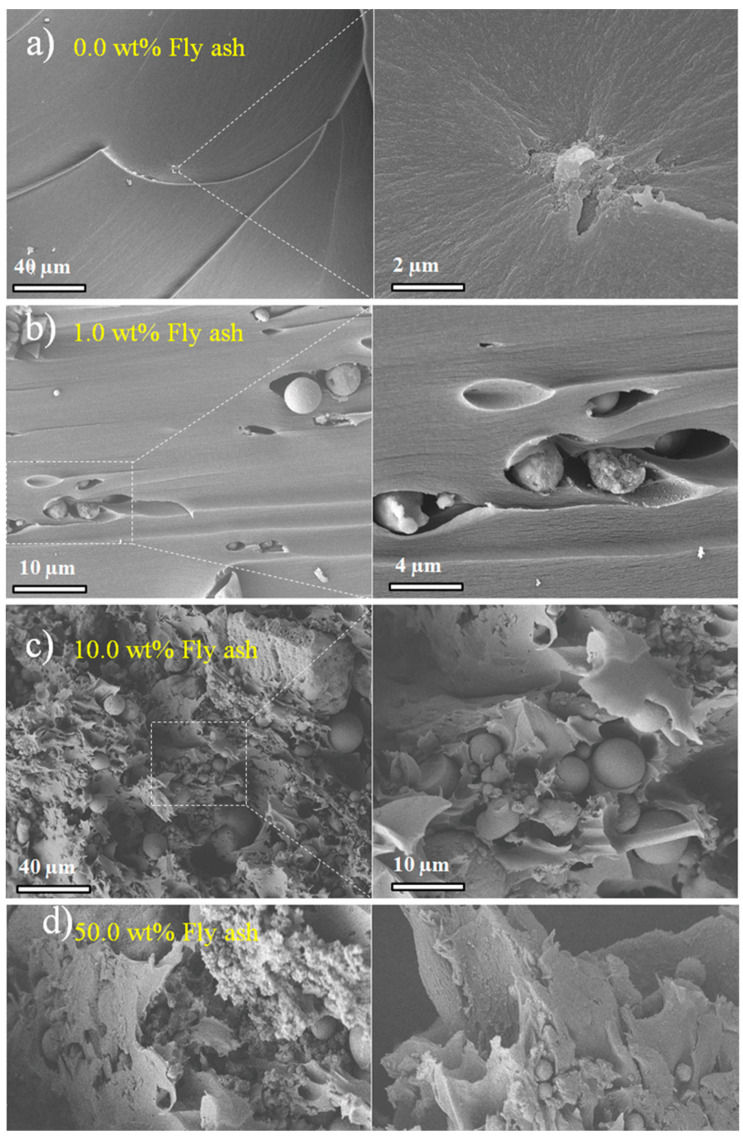
SEM micrographs of samples post-compression testing: (**a**) 0.0 wt.%; (**b**) 1.0 wt.%; (**c**) 10.0 wt.%; and (**d**) 50.0 wt.% fly ash samples.

The failure surfaces of the high-particle-content samples obtained after compression tests are shown in Figure 4. The detachment particle-DCPD matrix is clear in Figure 4b, which also shows the stress direction. The presence of FA particles induces a noticeable shift in the failure mode, transitioning from a relatively clean and brittle mode, as shown in Figure 4a, to a more ductile mode, as depicted in Figure 4b. However, in cases where the particle content reaches 10.0 wt.% (Figure 4c) and 50.0 wt.% (Figure 4d), the particle concentration becomes so substantial that visualizing crack growth becomes exceedingly challenging. In fact, this high particle concentration fosters particle agglomeration, leading to the formation of voids due to inadequate resin impregnation.

**Figure 5:**
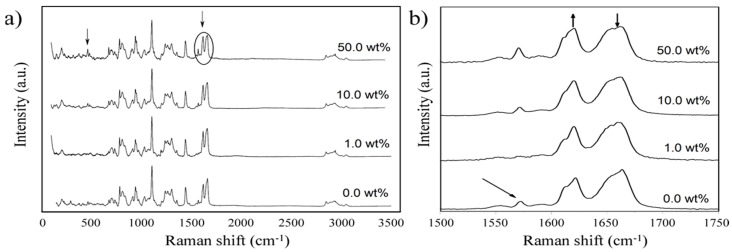
Raman spectroscopic analysis of fabricated composite variants. Raman spectroscopic analysis for (**a**) fabricated composite variants, (**b**) magnification of part (**a**) between 1500 and 1750 cm^−1^. The arrows show the absorption band locations.

According to the most acceptable structure for DCPD [33], the cross-linked polymer in general contains a bicyclic norbornene unit linked to a double bond of the substituted ethylene group. Because this polymer lacks electron-rich or hydrogen-bonding groups, when it serves as the matrix in a composite material, interactions with the inorganic phase are primarily anticipated to occur through the double bonds (sp2 hybrid external orbitals). Figure 5 shows the Raman spectra obtained for different DCPD/FA ratios. Typical vibration bands can be observed for the polymer [34]; however, some notable spectral featured changes should be pointed out. The absorption band at 1568 cm^−1^ relating to the ν(C=C) stretching mode of the double bond in the bicyclic norbornene unit (3) almost disappeared at 1.0 wt.% of FA in the sample; then, at 10.0 wt.% of FA, it appeared again. The decrease at 1.0 wt.% of FA and then the recovery of the intensity at 50.0 wt.% can be explained as a saturation limit after certain reinforcing percentages. On the other hand, the band at 1660 cm^−1^ related to ν(C=C) stretch of the aliphatic double bond of the substituted ethylene group (see Figure 5b) decreased in intensity when compared to the band at 1612 cm^−1^, which could be attributed to the opening of the cyclopentene ring [35,36]. This decrease in intensity suggests a strong interaction of the inorganic reinforce phase with the double bond of the ethylene group, possibly blocking the structural vibrations. As depicted in Figure 4, the flywheels exhibit uniform dispersion within the cross-linked polymeric structure. It is conceivable that secondary interactions involving the polymer double bonds and these microparticles contribute to the attenuation of certain vibrational modes, as corroborated by Raman spectroscopy.

**Figure 6:**
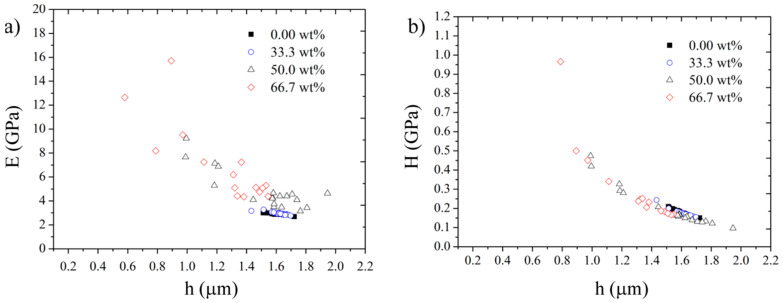
Nanoindentation test results: (**a**) elastic modulus and (**b**) nano-hardness.

The nanoindentation results are shown in Figure 6 and Table 3. For 0.0 and 1.0 wt.% of particle loading, dispersion of results is small, while the other samples do present a high dispersion in both stiffness and hardness. As can be seen, both the hardness and elastic modulus are not affected by 0.1 reinforcement level. On the other hand, for 10.0 and 50.0 wt.%, at 10 mN, the maximum depth and evaluated properties show a high numerical dispersion. This dispersion occurs because the indentations can be placed on top of the ash particles, which are harder and stiffer than the matrix. As a result, both hardness and stiffness tend to increase in the mean values (Table 3), which can be understood as a consequence of the particle reinforcement of the matrix. To obtain more accurate values for 10.0 and 50.0 wt.%, a higher maximum load must be applied, considering that the reduced modulus is converted to the elastic modulus by accounting for the elastic properties of the indenter.

**Figure 7:**
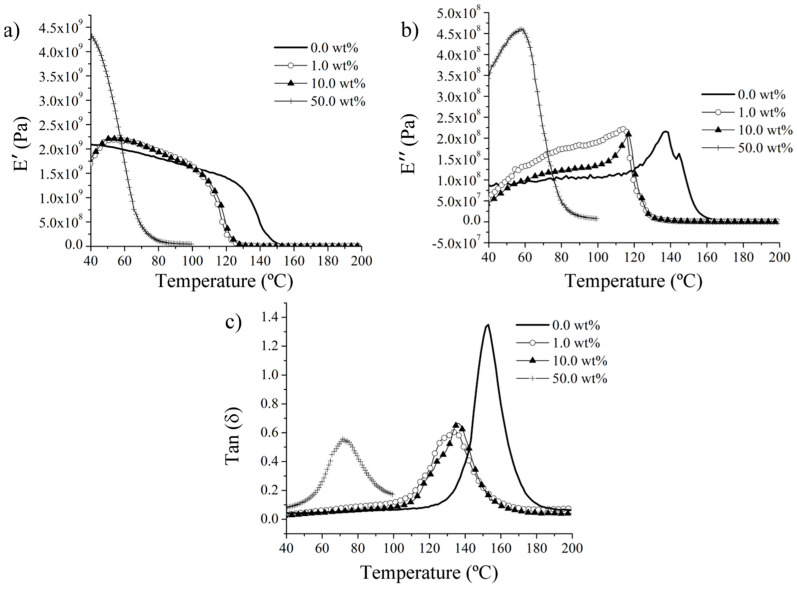
Dynamic mechanical analysis characteristics: (**a**) storage modulus (E′), (**b**) loss modulus (E″), and (**c**) tangent δ for fly ash samples at different loading levels of 0.0 wt.%, 1.0 wt.%, 10.0 wt.%, and 50.0 wt.%.

The observed shift in glass transition temperature (Tg) towards lower values is of paramount importance in the context of the authors’ proposed application of this versatile organic structure for shock mitigation. This shift offers a tailored approach to strategically relocate the maximum damping effect towards lower temperatures, being able to achieve room-temperature damping. Figure 7 effectively illustrates the influence of the incorporation of hollow spherical ash particles: Broadly speaking, the addition of spherical ash causes an overall increase in the elastic modulus (storage modulus). The presence of air trapped in both the hollow ash and the pores significantly increases the damping effect. At temperatures near room temperature, the tangent δ approaches zero for most materials, except for those with 50 wt.% reinforcement. Notably, although the higher reinforcement content results in a stiffer material, it also substantially amplifies the damping effect of the spheres, as evident in the reduction in the tangent δ and the shift of the damping peak toward lower temperatures with increasing reinforcement content. In particular, the temperature range up to the Tg increases the damping effect, as observed in the measurements of E″ and tangent δ.

**Figure 8:**
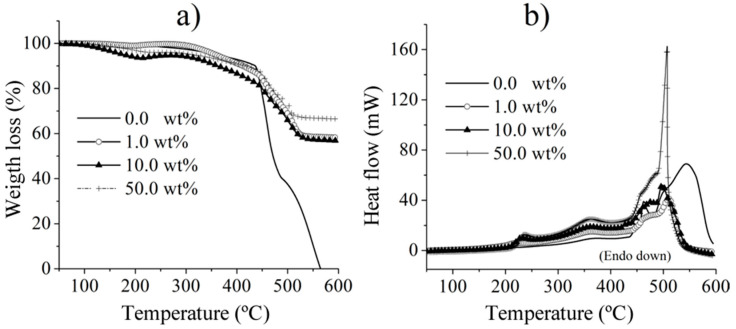
TGA findings: (**a**) weight loss and (**b**) heat flow.

From the storage modulus and tangent δ, FA decreases the glass transition temperature of the composite, which is important and allows applications at lower temperatures, with the potential to even modify this temperature to lower values. This is thought to be closely related to the curing peaks, which limits the released heat in the reaction, therefore affecting the thermomechanical behavior of the composites, and is complemented by the ceramic powders’ additional effects on thermal resistance. Finally, Figure 8 shows the TGA results for the fabricated composites, clearly revealing that as the ceramic particles increase, the thermal stability of the sample increases as well, when compared with the neat resin. Furthermore, there is minimal variation in weight loss among the samples containing 1.0, 10.0, and 50.0 wt.% of FA. This consistency can be attributed to the impact of the ceramic particles within the polymer matrix.

The findings presented above offer promising prospects for poly-DCPD and its composites, particularly in the realm of high-performance materials. This extends to applications in armor, military, and civil engineering. Specifically, composites incorporating ceramic fillers like fly ash have demonstrated the ability to extend both the setting time and, notably, the compressive strength. This effect is most pronounced at high particle loadings, such as 50 wt.%, offering the dual benefits of reducing composite costs and contributing significantly to the concept of material circularity [37]. Circular material practices aim to maximize a material’s use and lifespan across multiple cycles, thereby reducing environmental impacts and energy consumption. This approach aligns with the imperative to reduce CO_2_ emissions, optimize economics, and fulfill social responsibility, particularly in waste management [38].

Moreover, these high-loading formulations create opportunities for employing robocasting technology [36], also known as direct ink writing, for 3D printing. This extrusion-based technology leverages the high particle loading to control viscosity to the extent that the resin can be printed similarly to concrete [39]. Consequently, it can be applied in various contexts, such as infrastructure development, building construction, and emergency repair of large structures.

An unexplored avenue is the combination of this resin with other ceramic fillers to optimize mechanical and thermal properties, as well as to make use of residual ceramics, thus contributing to environmentally friendly practices in the ceramic industry and other green technologies. The material combinations investigated in this research open up possibilities for novel applications and innovative solutions.

## 4. Conclusions

This research underscores the utilization of cost-effective FA particles as a reinforcing agent within a poly-DCPD matrix, extending their potential applications from structural materials to impact-resistant components. Remarkably, the inclusion of up to 50 wt.% of FA particles resulted in exceptional compression performance. Furthermore, as the FA content increased within the poly-DCPD matrix, the composites exhibited enhanced thermal stability, a crucial attribute for numerous structural applications.

This investigation also revealed a remarkable interaction between poly-DCPD and FA particles. In particular, the introduction of a mere 1.0 wt.% of particles resulted in a significant decrease in the maximum cure temperature from 193 to 175 °C compared to the neat poly-DCPD sample. This phenomenon suggests a substantial impact of FA constituents, possibly stemming from their partial incorporation into the resin or subtle chemical modifications that may not be immediately evident through conventional chemical analysis. Among these constituents, calcium ions found in FA oxides appear to exert a particularly influential role.

Moreover, the compressive strength values of all composite samples exceeded 100 MPa, rendering this material a feasible solution for a wide spectrum of structural applications. These applications encompass lightweight materials like foams [40], impact-resistant materials [41], and construction components, where FA particles have traditionally found extensive use in cement and concrete formulations.

While poly-DCPD boasts excellent inherent mechanical properties, the incorporation of FA and similarly abundant, cost-effective particles demonstrates the capacity to significantly enhance thermomechanical strength without incurring substantial costs. Furthermore, this research opens up possibilities for incorporating ceramic waste powders into polymer resins, particularly for structural and impact-related applications. This approach aligns with sustainable practices by repurposing waste materials and enhancing the performance of composite materials for various industrial uses.

## Figures and Tables

**Table 1:** Fabricated sample variants.

Sample Reference	Composition (wt.%)	Sample Reference
	Fly Ash	59–63
0.0 wt.%	0	100
1.0 wt.%	1	99
10.0 wt.%	5	95
50.0 wt.%	50	50

**Table 2:** Range of chemical composition for fly ash class F.

Composition	Percentage
CaO	5–22
SiO_2_	59–63
Fe_2_O_3_	2–5
Al_2_O_3_	11–15

**Table 3:** Results of nanoindentation testing.

Sample Reference	Maximum Contact Depth µm	Elastic Modulus [GPa]	Hardness [GPa]
0.0 wt.%	1.58 ± 0.05	2.94 ± 0.10	0.19 ± 0.02
1.0 wt.%	1.62 ± 0.05	2.95 ± 0.12	0.17 ± 0.02
10.0 wt.%	1.52 ± 0.28	4.90 ± 1.65	0.21 ± 0.11
50.0 wt.%	1.24 ± 0.30	7.01 ± 3.33	0.44 ± 0.56

## Data Availability

The data presented in this study are available on request from the corresponding author.
